# Supplementary material for: The Systems Biology Research Tool: evolvable open-source software
Source: BMC Syst Biol. 2008 Jun 29;2:55. doi: 10.1186/1752-0509-2-55 (PMC2446383; doi:10.1186/1752-0509-2-55)
Supplement: Additional file 1 — SBRT Archive. An archive of the current version of the Systems Biology Research Tool. [file 1752-0509-2-55-S1.zip › sbrt-1.4.0/doc/developers_guide/api/sbrt/shell/io/fba/class-use/FluxIntervalInputFile.html]

Uses of Class sbrt.shell.io.fba.FluxIntervalInputFile


|  |  |  |
| --- | --- | --- |
| **Overview** \| **Package** \| **Class** \| **Use** \| **Tree** \| **Deprecated** \| **Index** \| **Help** | |  |
| PREV   NEXT | **FRAMES**    **NO FRAMES**     **All Classes** |  |


---


## **Uses of Class sbrt.shell.io.fba.FluxIntervalInputFile**

| Packages that use FluxIntervalInputFile | |
| --- | --- |
| **sbrt.shell.mng.fba** | Provides classes and interfaces for creating Flux Balance Analysis process managers. |

Uses of FluxIntervalInputFile in sbrt.shell.mng.fba

| Methods in sbrt.shell.mng.fba that return FluxIntervalInputFile | |
| --- | --- |
| `static FluxIntervalInputFile` | `IntervalVariationManager.getCvInputFile(ManagerInput<java.lang.String,java.lang.String> input, java.io.PrintWriter out, Fluxome fluxome)`             Returns the constraint variation input with the name denoted by `IntervalVariationManager.CV_FILE` in the provided manager input. |
| `static FluxIntervalInputFile` | `IntervalVariationManager.getCvInputFile(java.lang.String fileName, Fluxome fluxome)`             Returns the constraint variation input file with the provided name. |
| `static FluxIntervalInputFile` | `IntervalVariationManager.getIntervalInputFile(java.lang.String fileName, Fluxome fluxome)`             Returns the flux interval input file with the provided name. |
| `static FluxIntervalInputFile` | `IntervalVariationManager.getIntervalInputFile(java.lang.String fileName, Fluxome fluxome, MapFormat<LinearComb<java.lang.String>,Interval> mapFormat)`             Returns the flux interval input file with the provided name. |
| `static FluxIntervalInputFile` | `IntervalVariationManager.getSvInputFile(ManagerInput<java.lang.String,java.lang.String> input, java.io.PrintWriter out, Fluxome fluxome)`             Returns the space variation input with the name denoted by `IntervalVariationManager.FLUX_INTERVAL_FILE` in the provided manager input. |

---


|  |  |  |
| --- | --- | --- |
| **Overview** \| **Package** \| **Class** \| **Use** \| **Tree** \| **Deprecated** \| **Index** \| **Help** | |  |
| PREV   NEXT | **FRAMES**    **NO FRAMES**     **All Classes** |  |


---
